# Antibacterial efficacy and mechanism of action of *VTC3*-knockout *Saccharomyces cerevisiae* against multidrug- resistant pathogenic bacteria

**DOI:** 10.3389/fmicb.2026.1937861

**Published:** 2026-10-21

**Authors:** Weibao Yang, Mengkun Li, Yu Zhang, Kan Hu, Bingjie Lu, Ailin Yuan, Zuobin Zhu, Ying Li

**Affiliations:** 1 School of Medical Technology, Xuzhou Medical University, Xuzhou, Jiangsu, China; 2 Suqian Zhongwu Hospital, Suqian, Jiangsu, China; 3 Department of Genetics, Xuzhou Medical University, Xuzhou, Jiangsu, China; 4 Jiangsu Key Laboratory of New Drug Research and Clinical Pharmacy, Department of Microbial and Biochemical Pharmacy, School of Pharmacy, Xuzhou Medical University, Xuzhou, Jiangsu, China

**Keywords:** *Escherichia coli*, multidrug-resistant bacterial infection, probiotics, *Staphylococcus aureus*, virulence, *VTC3-*KO *Saccharomyces cerevisiae*

## Abstract

**Introduction:**

Multidrug-resistant (MDR) bacterial infections are a global public health crisis, and traditional antibiotic therapies are becoming increasingly ineffective. Probiotics represent a promising strategy in this context.

**Methods:**

In this study, we examined the antibacterial activity and mechanism of action of *Saccharomyces cerevisiae* with *VTC3* gene knockout (*VTC3-*KO).

**Results:**

Growth curves, biofilm formation, antibacterial spectrum, and coculture experiments demonstrated that *VTC3-*KO *S. cerevisiae* effectively inhibited the growth of pathogens and significantly suppressed the biofilm formation of *Escherichia coli*, *Staphylococcus aureus*, and *Klebsiella pneumoniae*. Moreover, *VTC3-*KO *S. cerevisiae* exhibited tolerance to the gastrointestinal environment, with enhanced auto and coaggregation with pathogens. In *in vivo* culture of *Galleria mellonella* and in a mouse infection model, *VTC3-*KO *S. cerevisiae* significantly improved host survival, alleviated intestinal tissue damage, and reduced inflammatory cell infiltration.

**Discussion:**

Mechanistic studies suggest that the observed antimicrobial effect may be attributable to the production of acidic substances. These findings demonstrate that *VTC3-*KO *S. cerevisiae* possesses dual antibacterial and probiotic functions, providing a sustainable alternative to traditional antibiotics for treating MDR infections.

## 1 Introduction

Multidrug-resistant (MDR) bacterial infections have become a global public health crisis, and the World Health Organization has ranked antibiotic resistance as one of the most serious public health threats of the 21st century. According to a study (GBD 2021 Antimicrobial Resistance Collaborators, 2024), 4.71 million deaths were attributed to MDR infections globally in 2021. By 2030, annual losses to global gross domestic product are projected to reach 1–3.4 trillion US dollars, and, by 2050, related healthcare costs will increase by an additional 1 trillion US dollars. Domestic surveillance data also indicate persistently high resistance rates among clinically common MDR bacteria, such as methicillin-resistant *S. aureus* (MRSA), carbapenem-resistant Enterobacteriaceae, and carbapenem-resistant *Acinetobacter* 42 *baumannii* (Sy et al., 2022). In 2024, the resistance rate of *A. baumannii* to carbapenems remained 43 above 64%, indicating a severe crisis (Guo et al., 2026).

The resistance mechanisms of MDR bacteria are complex and diverse, and include gene mutations, horizontal gene transfer, and biofilm formation that cause a marked decline in the efficacy of traditional antibiotics (Sy et al., 2022). Although increasing doses or the use of antibiotic combinations can temporarily control infections, these approaches frequently cause adverse effects and secondary and cross-infections as well as significantly increase mortality and healthcare costs (Javan et al., 2025). Although the development of combination therapies and novel antibiotics has advanced, the dilemma of resistance remains unresolved, with persistent challenges such as high costs and limited safety persist (Faridgohar, 2020; Javan et al., 2025). Therefore, developing safe, effective, and non-antibiotic interventions that do not readily induce resistance is crucial in the anti-infection field. Probiotics are live microorganisms that, when administered in adequate amounts, confer health benefits on the host (Gupta and Garg, 2009). They offer new strategies against MDR infections by modulating host immunity, inhibiting pathogen colonization, and repairing the intestinal barrier (Sy et al., 2022; Li et al., 2025; Wende et al., 2025).

Compared with traditional lactic acid bacteria, yeasts exhibit stronger environmental tolerance, a greater capacity to secrete various enzymes, and a unique cell-wall glucan structure, demonstrating superior probiotic potential in inhibiting pathogens, ameliorating inflammation, and treating antibiotic-associated diarrhea (Pais et al., 2020; Ding et al., 2025). Some S. cerevisiae strains inhibit pathogen growth and interfere with biofilm formation by secreting organic acids and antioxidant substances, with targets distinct from traditional antibiotics; thus, potentially circumventing bacterial resistance (Pais et al., 2020). Importantly, genetic engineering of S. cerevisiae has emerged as a highly effective strategy to uncover strains with enhanced antimicrobial activities. As demonstrated in our previous studies, targeted knockout of BTS-1 (Cong et al., 2025) and TAD-1 (Zhang et al., 2025) significantly enhanced the antibacterial effects of yeast against pathogens. Building on this foundation, we further targeted *VTC3*, a vacuolar membrane transport protein involved in polyphosphate (PolyP) synthesis and transport. PolyP plays a key role in microbial stress responses, virulence regulation, and biofilm formation, suggesting that the *VTC3*/PolyP pathway may be closely linked to the antibacterial functions of yeast (Fakruddin et al., 2017; Guan et al., 2023). However, the impact of *VTC3* knockout on the antibacterial activity of S. cerevisiae, particularly against clinical MDR bacteria, and the underlying molecular mechanisms remain largely unreported. Therefore, in this study, we aimed to investigate the *in vitro* and *in vivo* antibacterial activity and probiotic potential of *VTC3*-KO S. cerevisiae, elucidate the potential mechanisms against MDR bacteria, and provide experimental evidence and a theoretical basis for developing novel yeast probiotic formulations and clinical anti-infection therapies.

## 2 Materials and methods

### 2.1 Strains and growth conditions

The YKOC was obtained from Invitrogen (Carlsbad, CA, USA) in 2014 and stored at −80°C in Yeast extract peptone dextrose (YPD) medium with 20% (v/v) glycerol. YPD medium consisted of 1% (w/v) yeast extract (Oxoid Ltd., Basingstoke, UK), 2% (w/v) peptone (Oxoid Ltd., Basingstoke, UK), and 2% (w/v) glucose (Sinopharm, Beijing, China) dissolved in deionized water. *Saccharomyces boulardii* CNCM I-745 was purchased from Biocodex (France). *E. coli* ATCC 25922, E. coli mcr-1, S. aureus ATCC 29213, S. aureus ATCC 25923, methicillin-resistant *S. aureus, K. pneumoniae* ATCC 700603, *K. pneumoniae* 2118, *Pseudomonas aeruginosa* 27853, *Pseudomonas aeruginosa* 1554, *Salmonella typhimurium* SL1344, and *Acinetobacter baumannii* 21-1 were kindly provided by the Laboratory of the Affiliated Hospital of Xuzhou Medical University and routinely cultured in Luria–Bertani (LB) broth at 37°C. LB medium was prepared by dissolving LB broth powder (Sangon Biotech) in deionized water to a final concentration of 25 g/L.

### 2.2 Preparation of CFS from *S. cerevisiae*

*VTC3*-KO *S. cerevisiae*, the parental strain BY4743, and *Saccharomyces boulardii* CNCM I-745 were cultured at 30°C at 200 rpm for 16 h. After incubation, the cultures were centrifuged at 4,000 × *g* for 10 min at 4°C. The supernatants were filtered through 0.22 μm filters; then, 100 μL of each filtrate was spread onto YPD plates and incubated at 30°C for 24 h to confirm sterility (Kim et al., 2020; Jin et al., 2025)^.^ The CFS samples were stored at −80°C until use. BY4743 and *S. boulardii* CNCM I-745 CFS served as the control.

### 2.3 Establishing yeast growth curves

*VTC3-*KO *S. cerevisiae*, BY4743, and *S. boulardii* were cultured in YPD medium at 30°C at 200 rpm for 24 h. Every 2 h, 100-μL samples were taken and the OD_600_ was measured using a 100 microplate reader to construct growth curves (St John et al., 2020).

### 2.4 Effect of *VTC3*-KO CFS on bacterial growth curves

Five pathogenic bacteria, namely, *E. coli* ATCC 25922, *E. coli* mcr-1, *S. aureus* ATCC 29213, MRSA, *and K. pneumoniae* ATCC 700603, were grown overnight in log phase and cell numbers were adjusted to 1 × 10^6^ colony forming units (CFU) mL^–1^ in LB medium. Bacterial suspensions were mixed 1:9 (v/v) with either *VTC3-KO* CFS, BY4743 CFS, *S. boulardii* CNCM I-745 CFS, YPD medium, or sterile water. *S. boulardii* CNCM I-745 CFS was used as the positive control, and sterile water was used as the blank control. The initial optical density (OD)_600_ was recorded, and the mixtures were incubated at 37°C at 200 rpm. Every 2 h, 100-μL samples were taken to measure OD_600_. The values were recorded for plotting growth curves (Cong et al., 2025).

### 2.5 Antibacterial activity of *VTC3*-KO CFS against different bacteria

Eleven indicator strains (E. coli ATCC 25922, *E. coli* mcr- 1, S. aureus ATCC 29213, S. aureus ATCC 25923, MRSA, *K. pneumoniae* 700603, *K. pneumoniae* 2118, *P. aeruginosa* 27853, *P .aeruginosa* 1554, *S. typhimurium* SL1344, and *A. baumannii* 21- 1) were screened for susceptibility. In 96-well plates, 90 μL of CFS was mixed with 10 μL of bacterial suspension (1 × 10^8^ CFU mL^–1^). CFS from BY4743 and *S. boulardii* served as control groups. After incubation at 37°C for 18–24 h, the OD_600_ was measured (Zhang et al., 2025). Growth rate was calculated as (OD _sample_/OD _control_), and inhibition was defined as inhibition rate ≥ 80% (1 – growth rate ≥ 0.8).

### 2.6 Biofilm inhibition assay

The inhibition of biofilm formation by *VTC3-*KO CFS was quantitatively analyzed using crystal violet (CV) staining (López-García et al., 2024). *E. coli* ATCC25922, *E. coli* (*mcr-*1), *S. aureus* ATCC29213, MRSA, and *K. pneumoniae* 700603 were cultured to 1 × 10^6^ CFU mL^–1^ and inoculated into 96-well plates containing 25%, 50%, or 90% (v/v) CFS, with YPD medium as the control. After incubation at 37°C for 18–24 h, planktonic bacteria were removed and each well was gently washed twice with 150 μL of sterile water. Adherent biofilms were fixed (37°C, 30 min), stained with 150 μL of 0.1% CV for 30 min, washed twice, and destained with 150 μL of destaining solution containing 30% methanol and 10% glacial acetic acid. The OD_600_ was measured, and the inhibition rate was calculated as follows:


Biofilminhibitionrate(%)=(1-OD/treatment

OD)control×100

### 2.7 Determination of adhesion ability

Overnight cultures of pathogenic bacteria (*E. coli* ATCC 25922 and *S. aureus* ATCC 29213) were diluted with LB to 1 × 10^6^ CFU mL^–1^ and mixed 1:1 (v/v) with 50% *VTC3-KO* CFS or YPD. YPD served as the control. Aliquots (4 mL) were dispensed into glass tubes placed at 30°C and incubated at 37°C for 24 h. After incubation, planktonic bacteria were collected and retained. Adherent cells were gently washed twice with phosphate-buffered saline (PBS), then resuspended in fresh PBS after detachment. The OD_600_ of the adherent cell suspension (OD_adhered cell_) was measured. The total cell suspension (planktonic + adherent) was washed once and resuspended in PBS, and the OD_600_ of the cell mixture (OD _cell mixture_) was measured (Kim et al., 2020). Adhesion ability (%) was calculated as


Adhesionability(%)=(ODadheredcell/ODcellmixture)×100

### 2.8 Hydrophobicity assay

Overnight cultures of pathogenic bacteria (*E. coli* ATCC 25922 and *S. aureus* ATCC 29213) were diluted with LB to 1 × 10^6^ CFU mL^–1^ and mixed with 50% (v/v) *VTC3*-KO CFS or YPD, and then incubated at 37°C for 24 h. YPD served as the control. Cells were collected, washed twice with PBS, and resuspended at an OD_600_ of 0.50 ± 0.05 (OD _initial value_). For measuring cell-surface hydrophobicity, 1.2 mL of the cell suspension was mixed with 0.3 mL of n-octane and vortexed for 3 min. After standing for 15 min to allow phase separation, the OD_600_ of the aqueous phase (OD _treatment_) was measured (Rosenberg, 2006). Hydrophobicity (%) was calculated as


Hydrophobicity(%)=(1-ODinitialvalue/

ODtreatment)×100

### 2.9 Extracellular polysaccharide (EPS) production

The procedure was similar to that described in section “2.8 Hydrophobicity assay.” After incubation, cells were collected, washed twice with PBS, and resuspended in 1 mL of 0.9% saline. Then, 1 mL of 5% phenol and 1 mL of 5% sulfuric acid were added. The mixture was incubated in the dark for 1 h, and the 153 absorbance was measured at 490 nm (Zhang et al., 2020). The EPS content (%) was calculated as 154 follows:


EPSquantification(%)=(OD/treatmentOD)control×100.

### 2.10 Yeast–bacterial coculture model

To investigate whether live *VTC3-KO S. cerevisiae* and BY4743 inhibit pathogen growth, a yeast–bacteria coculture model was established. Overnight cultures of yeast and the pathogenic bacteria, *E. coli* ATCC 25922, *S. aureus* ATCC 29213, and *K. pneumoniae* 700603,were washed twice with PBS and resuspended in YPD or LB medium to a concentration of 1 × 10^7^ CFU mL^–1^. For coculture groups, 1 mL of yeast suspension was mixed with 1 mL of bacterial suspension (1:1, v/v). For bacterial monoculture, 1 mL of YPD was added. For blank control, 1 mL of sterile water was added. All tubes were incubated at 37°C at 200 rpm. At 16 h and 24 h, samples were taken and plated on LB agar containing 10 μg mL^–1^ amphotericin B to count viable bacteria (Liu et al., 2022).

### 2.11 Probiotic potential of *VTC3*-KO *S. cerevisiae*

(1) Tolerance to simulated gastrointestinal fluids

Simulated gastric fluid: PBS (pH 3.0) containing pepsin (3 mg mL^–1^). Simulated intestinal fluid: PBS (pH 8.0) containing trypsin (1 mg mL^–1^) and 0.3% bile salts. Both solutions were sterilized by filtration through 0.22 μm filters. *VTC3*-KO *S. cerevisiae* was cultured in YPD for 16 h (37°C, 200 rpm), centrifuged (4,000 × *g*, 4°C, 12 min), washed twice with PBS, and then resuspended in simulated gastric or intestinal fluid. The strains were incubated in gastric fluid for 1 h and 3 h, or in intestinal fluid for 2 h and 4 h (37 °C water bath). Viable cell counts were determined by using the dilution plating method (Rajkowska and Kunicka-Styczyńska, 2010).

(2) Autoaggregation assay

Overnight cultures of *VTC3*-KO *S. cerevisiae* and BY4743 were collected, washed twice with PBS, and resuspended in PBS to an OD_600_ of 0.5 (A_0_). The suspension was then allowed to stand at room temperature for 5 h, and the OD_600_ of the upper phase (A_t_) was measured (Tomičić et al., 2022). Autoaggregation (%) was calculated as follows:


Autoaggregation(%)=[1-(A/tA)0]×100.

(3) Coaggregation assay

Overnight cultures of pathogenic bacteria were collected (8,000 × g, 4°C, 10 min), washed twice with PBS, and resuspended to an OD_600_ of approximately 0.5 (A_1_). Yeast suspensions (*VTC3-KO S. cerevisiae* and BY4743) were prepared similarly (A_2_). Then, 2 mL of each yeast suspension was mixed with 2 mL of the corresponding bacterial suspension and incubated statically at 37°C for 5 h. The OD_600_ of the upper phase (A_3_) was measured (Tomičić et al., 2022). Coaggregation (%) was calculated as


Coaggregation(%)=[((A+1A)2/2-A)3/

((A+1A)2/2)]×100.

### 2.12 *G. mellonella* infection model

*G. mellonella* was used to evaluate the antibacterial activity of *VTC3*-KO *S. cerevisiae* against pathogens (Tsai et al., 2016). Yeast cells were collected, washed three times with PBS, and resuspended in PBS to prepare a live yeast suspension at a concentration of 5.0 × 10^8^CFU mL^–1^. For heat-inactivated yeast, 5 mL of the suspension was autoclaved at 121°C for 20 min, centrifuged at 8,000 × *g* for 10 min, washed three times, and resuspended in PBS, at a concentration of 5.0 × 10^8^CFU mL^–1^. The pathogenic bacteria were adjusted to 1.0 × 10^8^ CFU mL^–1^. *G. mellonella* were randomly divided into eight groups: one blank control (PBS buffer group), one control (single-pathogen group), three toxicity groups (CFS group, Alive group, Heat-killed group), and three treatment groups (CFS + pathogen group, alive + pathogen group, Heat-killed + pathogen group). All groups were kept in the dark at 37°C, and *G. mellonella* survival was recorded for 5 days. On day 5 post-infection, *G. mellonella* larvae from each group were homogenized, serially diluted, and spread plated to enumerate the bacterial load in vivo, followed by graph plotting.

### 2.13 Mouse model of enteritis

An enteritis model of mice infected with *S. typhimurium* SL1344 was established (Cong et al., 2025; Li et al., 2026). Specific-pathogen-free grade male C57BL/6J mice were purchased from Jiangsu Jicui Pharmachem Biotechnology Co., (Nanjing, China), Laboratory Animal Production License No. SCXK (Su) 2018-0008; Laboratory Animal Use License No. SYXK (Su) 2020-0048 (barrier environment). All animal experimental protocols were approved by the Animal Ethics Committee of Xuzhou Medical University.

*VTC3-*KO *S. cerevisiae* and BY4743 were harvested, washed twice with PBS, and resuspended at 5.0 × 10^8^ CFU mL^–1^. Thirty-six mice were randomly allocated into three groups (*n* = 12 each) and gavaged once daily for 7 days with 0.1 mL of: *VTC3-*KO *S. cerevisiae* suspension, BY4743 suspension, 0.9% saline (control). Individual animals were identified by tail markings; their general appearance and body weight were recorded daily.

Each of the three treatment groups was split into two sub-cohorts (*n* = 6). Sub-cohort 1 (5-day infection study): Mice were challenged orally with 1 × 10^7^ CFU mL^–1^ of S. typhi and weighed daily. On day 5, the animals were euthanized; the ileum, colon, and caecum were aseptically removed. The plate count method was used to enumerate colonies of S. typhi. Sub-cohort 2 (survival study): After an identical challenge, mice were monitored for 10 days, and their survival was recorded.

The spleens of the three groups of mice were weighed and photographed, and the spleen index was calculated. We used the following equation to calculate the spleen index (%): spleen index (%) = (spleen weight/body weight) × 100. Three groups of mouse colon tissues were taken to measure the length and were photographed. Then, 2-cm tissue segments were harvested from the terminal ileum for hematoxylin-eosin staining (H&E) to evaluate villus structure, the degree of inflammatory infiltration, and epithelial integrity.

Quantitative Real-Time PCR: Total RNA was extracted from the ileal segments with TRIzol (TIANGEN, Beijing, China) and quantified on a NanoDrop-1000 spectrophotometer (Thermo Fisher, Waltham, MA, USA); only samples with A260/A280 ≥ 1.9 were processed. cDNA was synthesized using a reverse-transcription kit (Sangon Biotech, Shanghai, China). Quantitative PCR was performed in a Light Cycler 480 (Roche, Basel, Switzerland) with Universal SYBR qPCR Master Mix (Biosharp, Hefei, China) and gene-specific primers (Sangon Biotech). Transcript levels of IL-6, IL-10, TNF-α, occludin, claudin-1, and ZO-1 were normalized to β-actin and expressed as 2^(–ΔΔCt)^ values.

### 2.14 Mebabolomic analysis

*VTC3-*KO *S. cerevisiae* and BY4743 were cultivated in parallel (YPD, 30°C, 200 rpm, 16 h; six biological replicates were set for each strain). The cultures were precooled on ice, centrifuged at 4000 × *g* and 4 °C for 12 min, and the supernatant was immediately filtered through a 0.22 μm sterile syringe filter. CFS were snap-frozen in liquid nitrogen and shipped on dry ice to Shanghai Majorbio Bio-Pharm Technology Co., Ltd., (Shanghai, China) for untargeted Liquid chromatography-tandem mass spectrometry (LC-MS/MS)-based metabolomic analysis. Upon receipt of samples by the company, sample pretreatment was performed as follows: Briefly, 200 μL of sample was transferred into a 1.5 mL centrifuge tube, followed by the addition of 800 μL methanol-acetonitrile (1:1, v/v) extraction solution containing four internal standards including L- 2-chlorophenylalanine (0.02 mg/mL). The mixture was vortexed for 30 s, subjected to low-temperature ultrasonic extraction at 5 °C and 40 kHz for 30 min, and incubated at −20°C for 30 min. Subsequently, centrifugation was performed at 4°C and 13000 × *g* for 15 min. The supernatant was collected and dried under nitrogen gas, then reconstituted with 120 μL acetonitrile-water (1:1) reconstitution solution. After vortexing for 30 s, the sample underwent low-temperature ultrasonic extraction at 5°C and 40 kHz for 5 min, followed by centrifugation at 4 °C and 13000 × *g* for 10 min. The resulting supernatant was transferred into an autosampler vial with an insert for instrumental analysis. Then, 20 μL of supernatant from each individual sample was pooled to prepare samples for quality control.

Detection was performed using a Thermo Fisher UHPLC- Q Exactive HF- X ultra- high-performance liquid chromatography coupled with Fourier-transform mass spectrometry system. Chromatographic separation was achieved on a Waters ACQUITY UPLC HSS T3 column (100 mm × 2.1 mm i.d., 1.8 μm). Mobile phase A consisted of 95% water and 5% acetonitrile containing 0.1% formic acid; mobile phase B comprised 47.5% acetonitrile, 47.5% isopropanol and 5% water. The injection volume was 3 μL, and the column temperature was maintained at 40 °C. Samples were ionized via electrospray ionization, and mass spectrometric signals were acquired under positive and negative ion modes (Zhang et al., 2025; Spaggiari et al., 2026).

Quality Control (QC) samples were prepared by pooling equal volumes of supernatants from all test samples and subjected to identical pretreatment and detection procedures as the experimental samples. One QC sample was injected for every 5–15 test samples during instrumental acquisition to evaluate the stability of the overall analytical workflow.

### 2.15 Identification of antibacterial components

To determine which components in *VTC3-*KO CFS are responsible for the antibacterial activity against *E. coli* and *S. aureus*, pH neutralization, enzyme treatment, and heat-treatment experiments were performed (Liu H. et al., 2023). The pH of untreated CFS was measured. Aliquots were adjusted to pH 2–7 using 0.1 M HCl or 0.1 M NaOH. CFS was also mixed with pepsin, trypsin, proteinase K, or papain and incubated at 37 °C for 2 h. Additionally, CFS was heated at 60 °C, 80 °C, or 100°C for 1 h. After treatment, the CFS was mixed with the bacterial suspension (final concentration 1 × 10^6^ CFU mL^–1^) at a ratio of 9:1 (v/v) and incubated at 30°C for 24 h. Growth inhibition was quantified by measuring OD_600_.

### 2.16 Statistical analysis

All experiments were performed in triplicates and repeated thrice on different days. GraphPad Prism 10 software was used for statistical analysis and graphing. Statistical significance was determined using independent samples t- test or one- way analysis of variance. Differences were considered significant at *p < 0.05, **p < 0.01, ***p < 0.005; ns indicated no significant difference.

## 3 Results

### 3.1 Knockout of *VTC3* does not substantially affect *S. cerevisiae* proliferation

In this study, disruption of the *VTC3* gene caused no detectable growth defect: the growth curves for *VTC3-*KO *S. cerevisiae* and BY4743 over 24 h were nearly identical (Figure 1), indicating that loss of *VTC3* does not significantly impair yeast growth under standard laboratory conditions.

**FIGURE 1:**
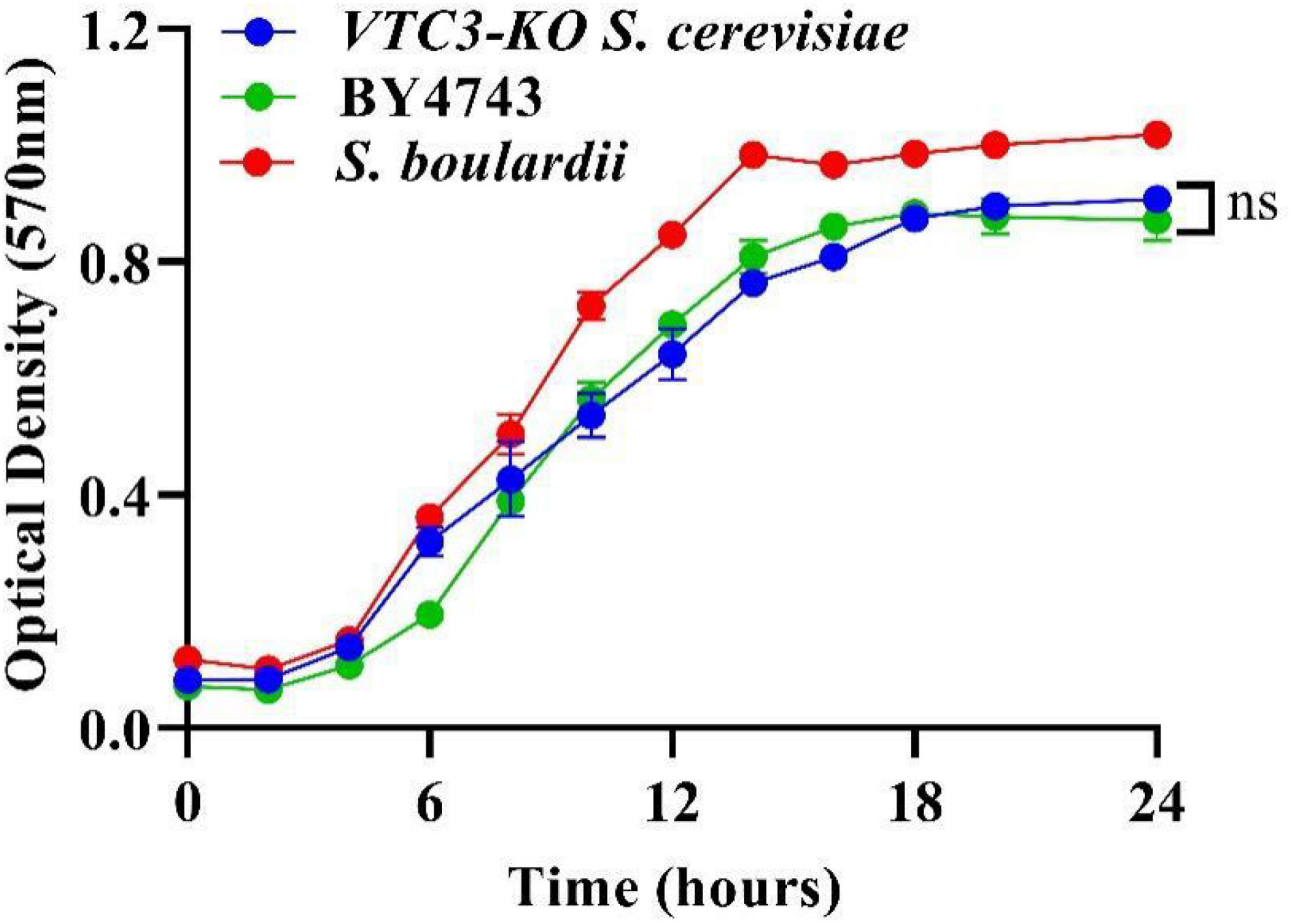
Growth curves of *VTC3-*KO *S. cerevisiae*, BY4743, and *S. boulardii*. Deletion of *VTC3* did not inhibit yeast growth; ns indicates no significant difference.

### 3.2 *VTC3-KO S. cerevisiae* CFS inhibits the growth of *E. coli*, *S. aureus* and *K. pneumoniae*.

Growth curve analysis showed that *VTC3-*KO *S. cerevisiae* CFS exhibited significant antibacterial activity. Compared with the untreated control, treatment with 90% (v/v) *VTC3-*KO *S. cerevisiae* CFS markedly delayed the transition from lag phase to log phase, reduced the maximum specific growth rate (μmax), and decreased total bacterial counts for *E. coli*, *S. aureus* and *K. pneumoniae*. Although BY4743 CFS also showed some antibacterial effects, these effects were weaker, confirming the contribution of *VTC3* deletion to antibacterial activity. To rule out nutrient limitation, parallel controls with sterile water and complete YPD medium were included. No significant differences in growth parameters were observed between these two extreme controls (Figures 2A–E), demonstrating that the observed antibacterial effect resulted from bioactive metabolites rather than from carbon or nitrogen depletion.

**FIGURE 2:**
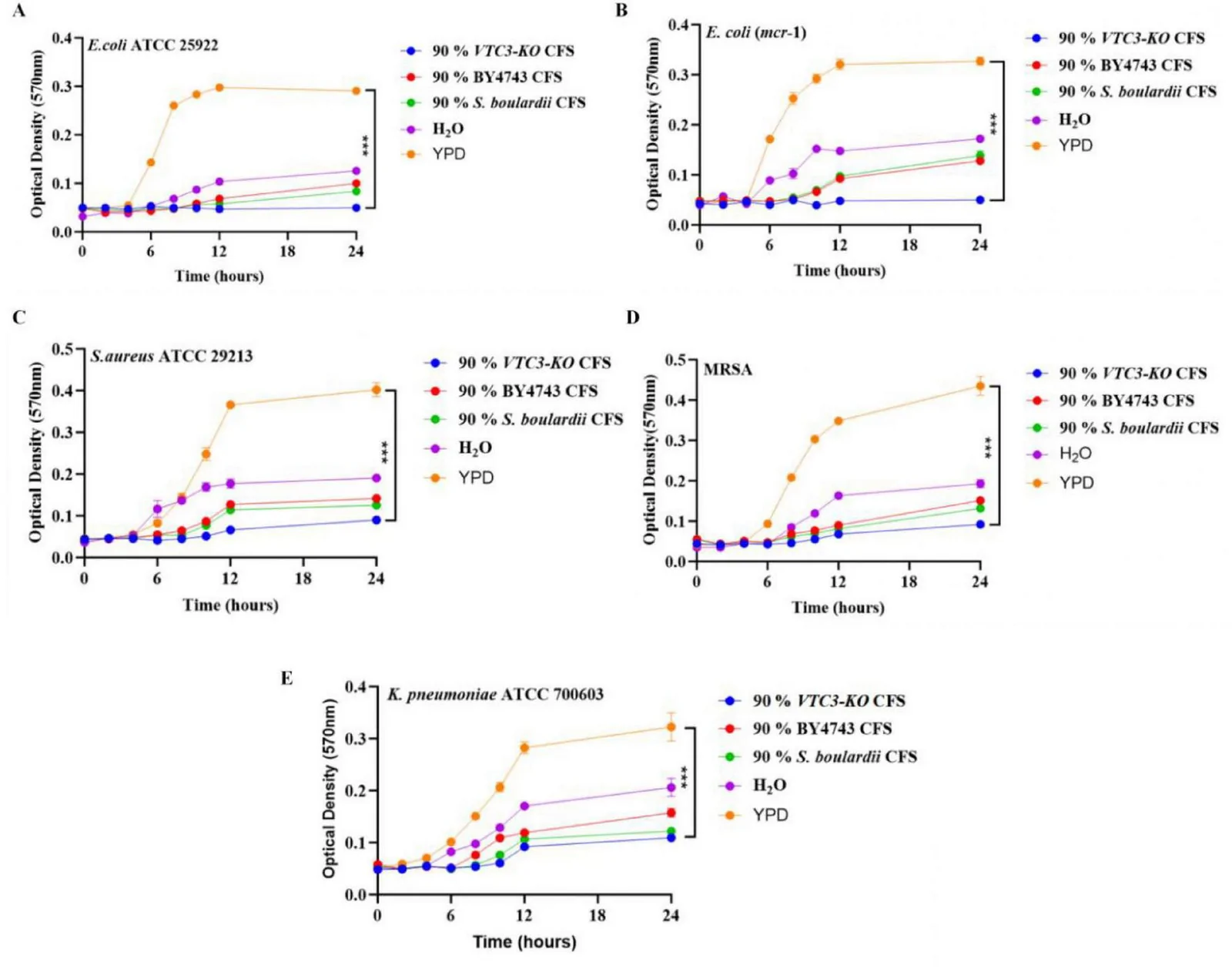
Growth curves of *E. coli* **(A,B)**, *S. aureus* **(C,D)**, and *K. pneumoniae* **(E)** under different treatments. Data are shown as mean ± standard deviation (SD). ***p < 0.001.

### 3.3 *VTC3-KO* CFS inhibits the growth of multiple pathogenic bacteria

Inhibition experiments against the various bacteria showed that *VTC3-*KO CFS had good antibacterial activity against common Gram-negative (*E. coli* ATCC25922) and Gram-positive bacteria (*S. aureus* ATCC29213 and *S. aureus* ATCC25923) as well as clinical MDR isolates (MRSA, *K. pneumoniae* ATCC700603 and *A. baumannii* 21-1) (Table 1). Moreover, the antibacterial activity of *VTC3-*KO CFS was significantly higher than that of BY4743 and *S. boulardii* CFS, confirming that *VTC3-*KO *S. cerevisiae* confers stronger broad- spectrum antibacterial capability.

**TABLE 1:** Bacterial inhibition profile of yeast CFS (growth rate %).

Strains	BY4743	*VTC3-*KO *S. cerevisiae*	*S. boulardii*
*E. coli* ATCC25922	49.37% ± 2.58%	18.31% ± 1.48%***	30.02% ± 4.61%
*E. coli* (*mcr*-1)	53.94% ± 0.60%	21.04% ± 0.39%***	58.58% ± 2.05%
*S. aureus* ATCC29213	78.49% ± 13.67%	37.91% ± 3.10%**	64.07% ± 3.11%
*S. aureus* ATCC 25923	55.83% ± 9.89%	27.69% ± 2.32%**	71.58% ± 1.08%
MRSA	68.41% ± 2.05%	37.04% ± 0.84%***	55.77% ± 2.65%
*K. pneumoniae* ATCC700603	38.78% ± 11.47%	30.54% ± 1.67%^ns^	52.34% ± 2.08%
*K. pneumoniae* 2118	27.77% ± 3.47%	19.41% ± 2.32%^ns^	29.75% ± 3.84%
*P. aeruginosa* 27853	14.50% ± 0.82%	15.61% ± 2.58%^ns^	9.83% ± 1.10%
*P. aeruginosa* 1554	27.36% ± 3.28%	3.70% ± 0.5%***	14.43% ± 1.41%
*S. typhimurium* SL1344	62.30% ± 1.59%	21.01% ± 2.95%***	64.09% ± 12.12%
*A. baumannii* 21-1	21.46% ± 6.08%	1.74% ± 0.03%**	18.79% ± 1.00%

Lower growth rate indicates stronger inhibition,

**p < 0.01;

***p < 0.001; ns, not significant.

### 3.4 *VTC3*-KO CFS inhibits biofilm formation

Crystal violet (CV) staining results demonstrated that treatment with 25%, 50%, and 90% (v/v) S. cerevisiae *VTC3*- KO CFS for 24 h resulted in dose-dependent inhibition of biofilm formation in *E. coli* ATCC 25922, *E. coli* (mcr- 1), *S. aureus* ATCC 29213, MRSA, and *K. pneumoniae* 700603 (Figure 3). The inhibition rates of 90% (v/v) S. cerevisiae *VTC3*- KO CFS against biofilm formation of *E. coli* ATCC 25922, *E. coli* (mcr- 1), S. aureus ATCC 29213, MRSA, and *K. pneumoniae* 700603 were 86.31%, 85.29%, 83.61%, 87.44%, and 83.05%, respectively. Collectively, these findings indicate that *S. cerevisiae VTC3*- KO CFS is capable of inhibiting biofilm formation by pathogenic bacteria.

**FIGURE 3:**
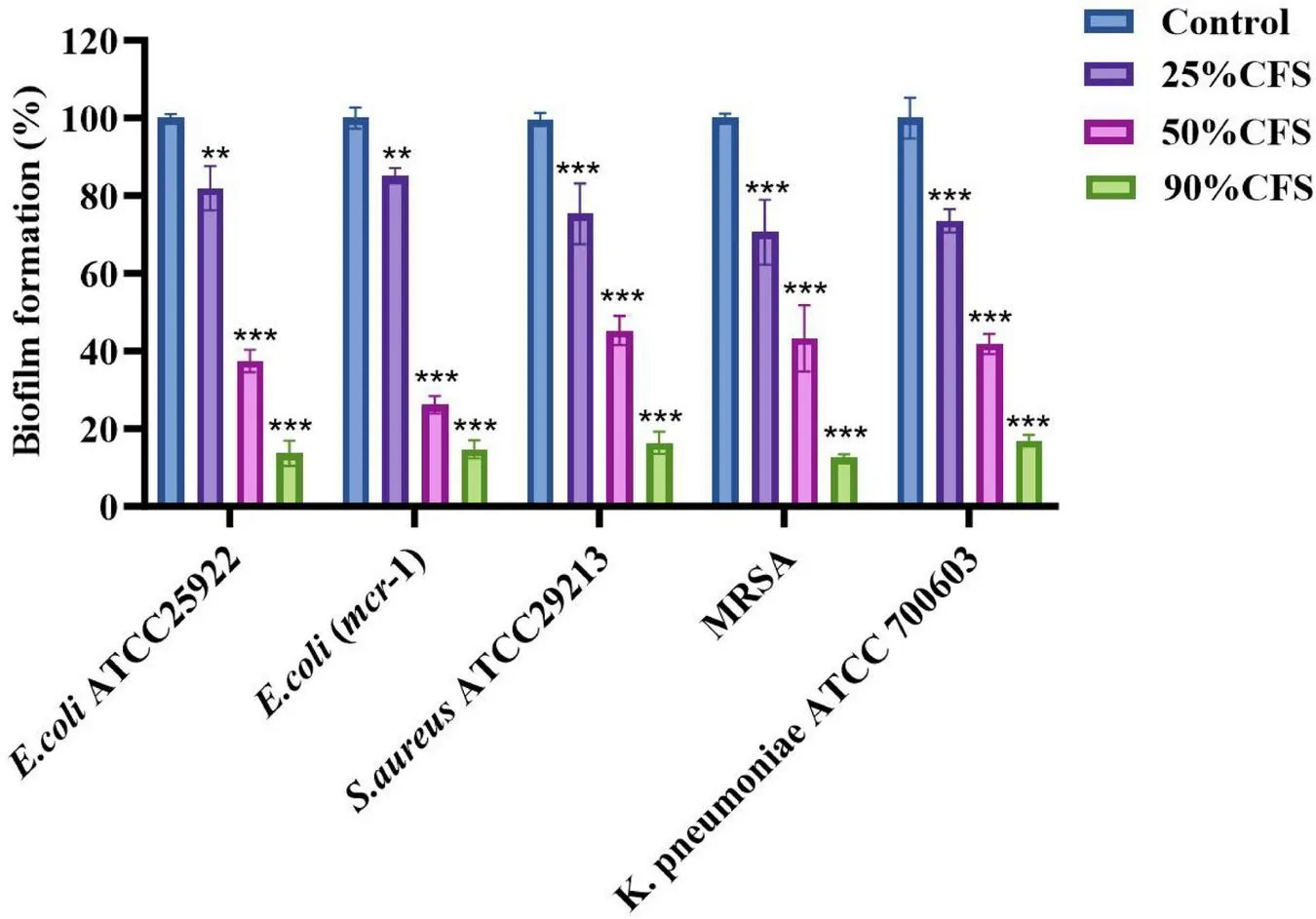
Effect of *VTC3-*KO cell-free supernatant (CFS) on biofilm formation by various bacterial strains. Data are shown as mean ± standard deviation (SD). **p < 0.01; ***p < 0.001.

### 3.5 *VTC3*-KO CFS reduced the adhesion, hydrophobicity, and EPS production of *E. coli* ATCC25922 and *S. aureus* ATCC29213

The results showed that after treatment with 50% (v/v) CFS, the adhesion ability of *E. coli* ATCC25922 and *S. aureus* ATCC29213 decreased by 29.45% and 37.89%, respectively (Figure 4A). The hydrophobicity of *E. coli* ATCC25922 and S. aureus ATCC29213 decreased by 13.05% and 16.28%, respectively (Figure 4B), and EPS production decreased by 22.61% and 16.57%, respectively (Figure 4C).

**FIGURE 4:**
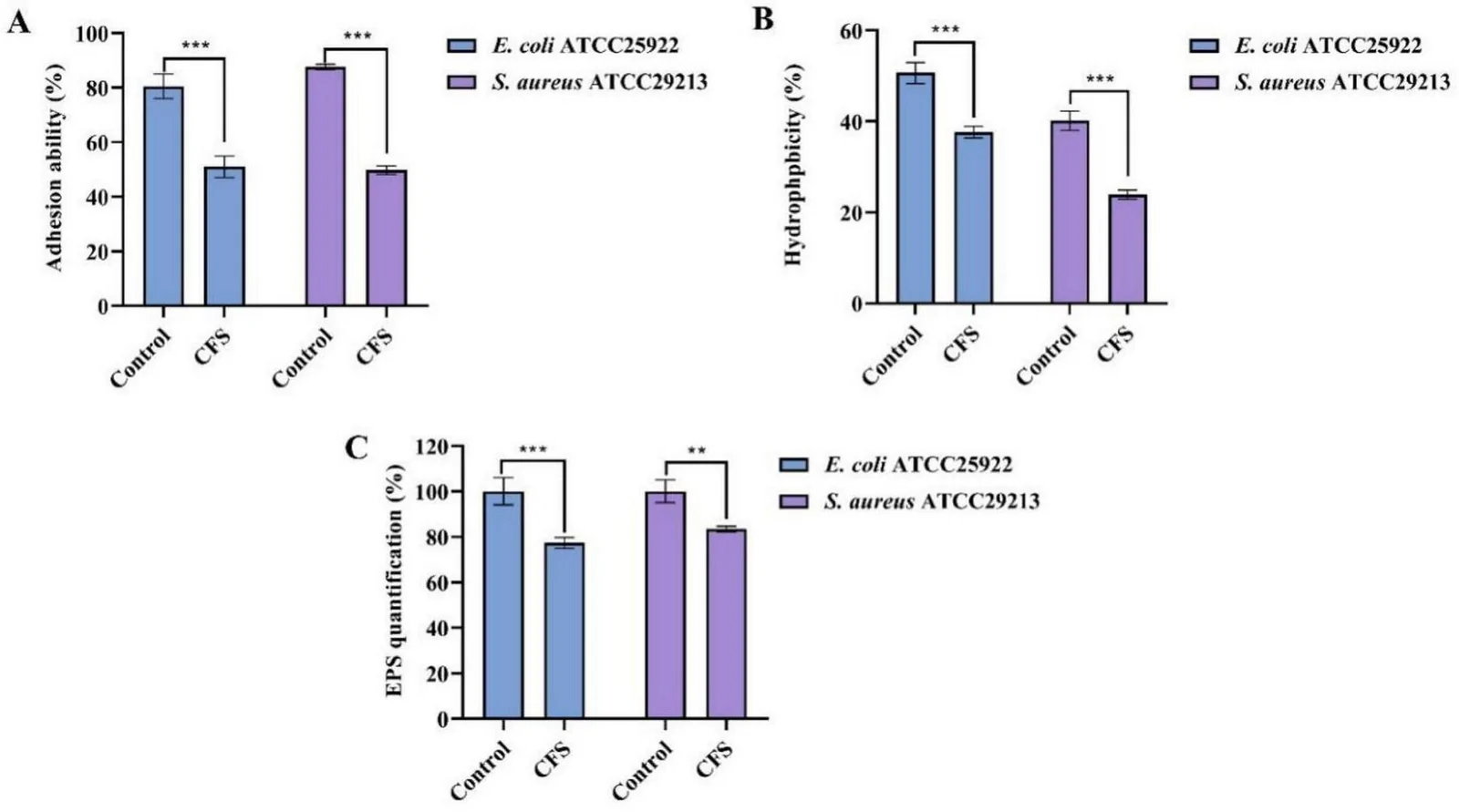
Effect of *VTC3-*KO cell-free supernatant (CFS) on the adhesion ability, surface hydrophobicity, and extracellular polysaccharide (EPS) production of *E. coli* and *S. aureus.* **(A)** CFS reduced adhesion ability. **(B)** CFS reduced surface hydrophobicity. **(C)** CFS reduced EPS production. Data are shown as mean ± standard deviation (SD). **p < 0.01; ***p < 0.001.

### 3.6 *VTC3*-KO S. cerevisiae inhibits the growth of pathogenic bacteria

A bacterial–yeast coculture model was used to verify whether live *VTC3*-KO S. cerevisiae could inhibit the growth of *E. coli* ATCC25922, *S. aureus* ATCC29213, and *K. pneumoniae* ATCC 700603. The results showed that compared with mono- culture in YPD liquid medium or H_2_O, *E. coli* ATCC25922, S. aureus ATCC29213, and *K. pneumoniae* ATCC 700603 cocultured with live *VTC3*KO S. cerevisiae showed significantly reduced cell numbers after 16 and 24 h. After 16 h of coculture, the inhibition rates of *VTC3*-KO S. cerevisiae against *E. coli* ATCC25922, *S. aureus* ATCC29213, and K. pneumoniae ATCC 700603 were 48.44% (1.1 × 10^9^ CFU mL^–1^), 89.88% (1.15 × 10^8^ CFUmL^–1^), and 45.77% (1.03 × 10^9^ CFU mL^–1^), respectively; after 24 h, the inhibition rates were 49.21% (1.07 × 10^9^ CFU mL^–1^), 89.81% (1.47 × 10^8^ CFU mL^–1^), and 45.07% (1.04 × 10^9^ CFU mL^–1^), respectively. Moreover, no significant difference in bacterial growth was observed between the H_2_O and YPD groups, indicating that bacterial proliferation was independent of nutrient richness (Figure 5).

**FIGURE 5:**
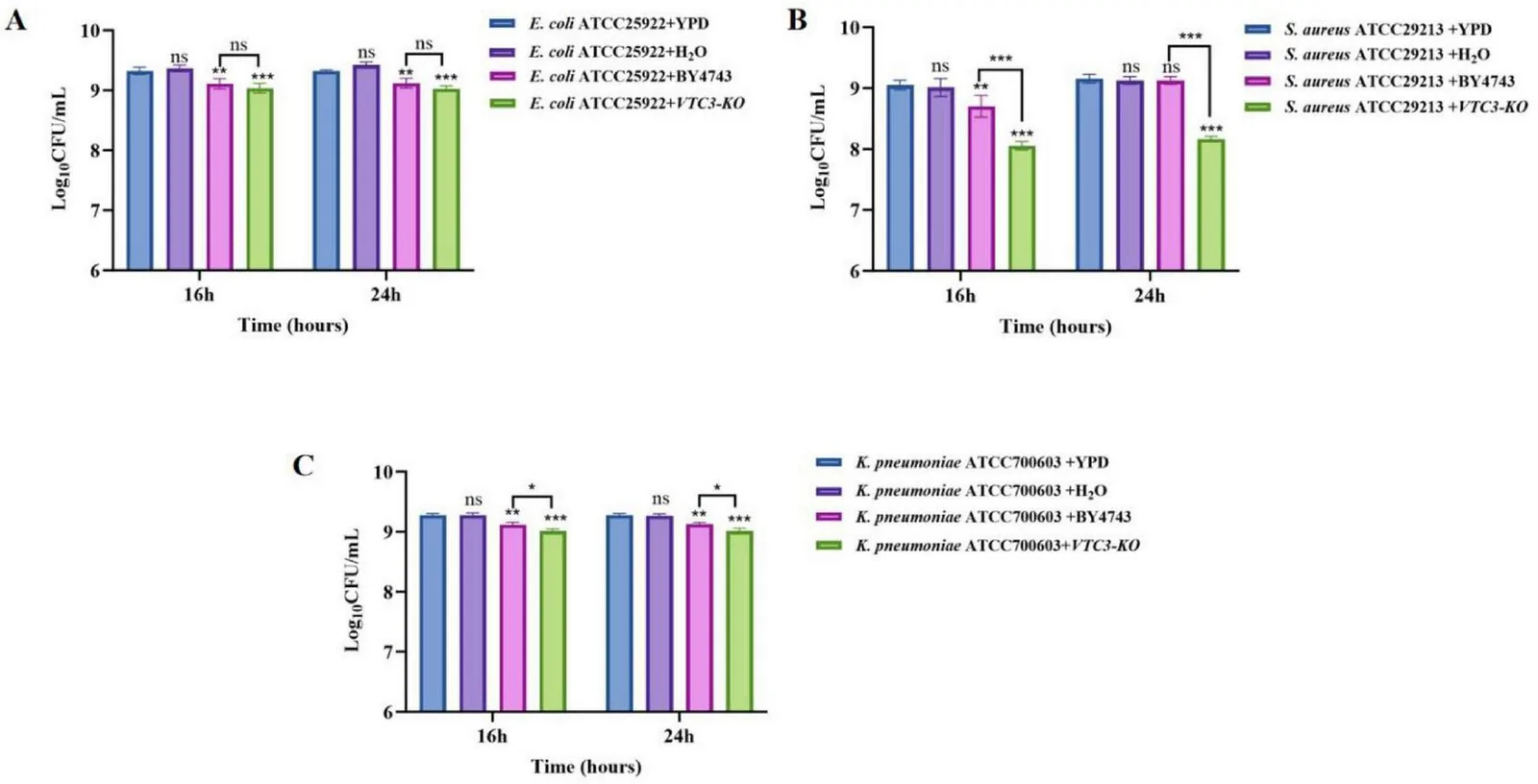
Coculture of *VTC3*-KO *S. cerevisiae* with pathogenic bacterial strains. **(A)** Coculture with *E. coli* ATCC25922. **(B)** Coculture with *S. aureus* ATCC29213. **(C)** Coculture with *K. pneumoniae* ATCC 700603. Data are shown as mean ± standard deviation (SD). ns, not significant; *p < 0.05;**p < 0.01; ***p < 0.001.

### 3.7 *VTC3*-KO *S. cerevisiae* possesses excellent probiotic properties

#### 3.7.1 Good tolerance to simulated gastric and intestinal fluids

For probiotics to colonize in the gastrointestinal tract and exert their effects, they must tolerate the digestive environment. Therefore, we examined the tolerance of *VTC3-*KO *S. cerevisiae* to simulated gastric and intestinal fluids. After 1 h in simulated gastric fluid at pH 3.0, the survival rate of *VTC3-*KO *S. cerevisiae* was as high as 82.13%; after 3 h, it remained above 75% (Figure 6A). Similarly, after 4 h in simulated intestinal fluid, the survival rate was 65.17% (Figure 6B). These results indicate that *VTC3-*KO *S. cerevisiae* can tolerate upper digestive tract stress and has the potential for *in vivo* colonization.

**FIGURE 6:**
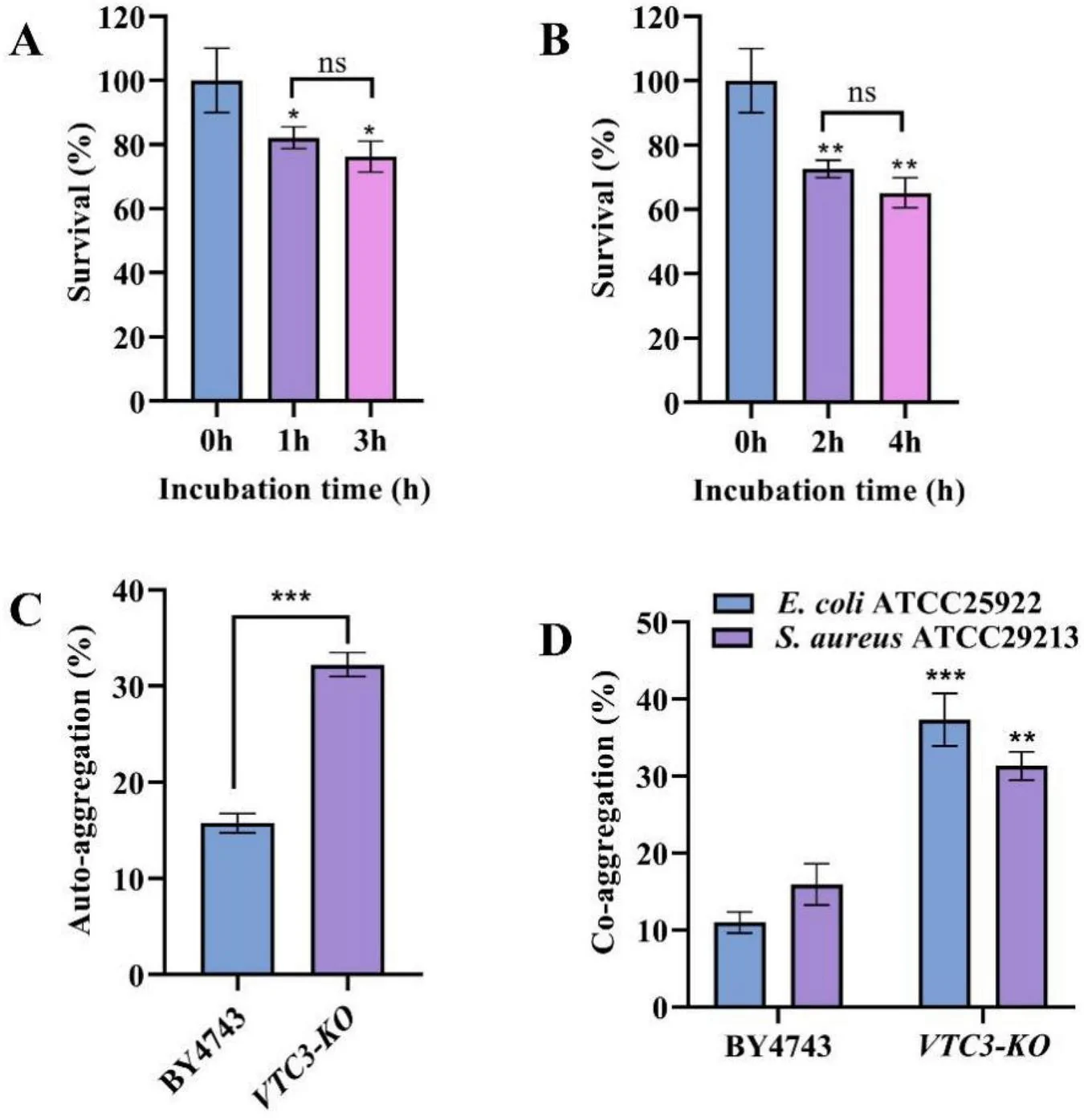
*VTC3-*KO *S. cerevisiae* tolerates simulated gastrointestinal stress and exhibits excellent aggregation properties. **(A)** Survival rates in simulated gastric fluid after 1 h and 3 h. **(B)** Survival rates in simulated intestinal fluid containing 0.3% bile salts after 2 h and 4 h. **(C)** Autoaggregation of *VTC3-*KO *S. cerevisiae* and BY4743. **(D)** Coaggregation of *VTC3-*KO *S. cerevisiae* and BY4743 with *E. coli* and *S. aureus*. Data are shown as mean ± SD. ns, not significant;*p < 0.05; **p < 0.01; ***p < 0.001.

#### 3.7.2 Increased auto- aggregation and coaggregation with pathogens

The results showed that *VTC3-*KO *S. cerevisiae* exhibited 16.48% higher auto- aggregation than BY4743 (Figure 6C) and formed significantly denser aggregates with pathogens, with coaggregation rates increased by 26.31% and 15.4% for *E. coli* and *S. aureus*, respectively (Figure 6D). These data suggest that *VTC3-*KO *S. cerevisiae* can establish close contact with target pathogens, thereby promoting the local release of antimicrobial metabolites in the intestinal lumen.

### 3.8 *In vivo* antibacterial therapeutic effect of *VTC3*-KO *S. cerevisiae*

#### 3.8.1 *VTC3*-KO *S. cerevisiae* prolongs the survival of *G. mellonella*

This model was used to evaluate the toxicity and antibacterial effect of *VTC3-*KO *S. cerevisiae*. Compared with the control group, treatment of healthy larvae with live yeast, heat- inactivated yeast, or CFS did not significantly change survival over 5 days, confirming the low toxicity of *VTC3-*KO *S. cerevisiae* (Figure 7A). In the infection model, live yeast and CFS significantly prolonged the survival of larvae infected with *E. coli* or *S. aureus*, whereas heat- inactivated yeast provided no protection (Figures 7B,D). Bacterial load measurement also showed that live yeast and CFS effectively reduced the bacterial load in larvae (Figures 7C, E). These data indicate that the antibacterial effect is mediated by metabolites actively secreted by live yeast, rather than by yeast cell structural components.

**FIGURE 7:**
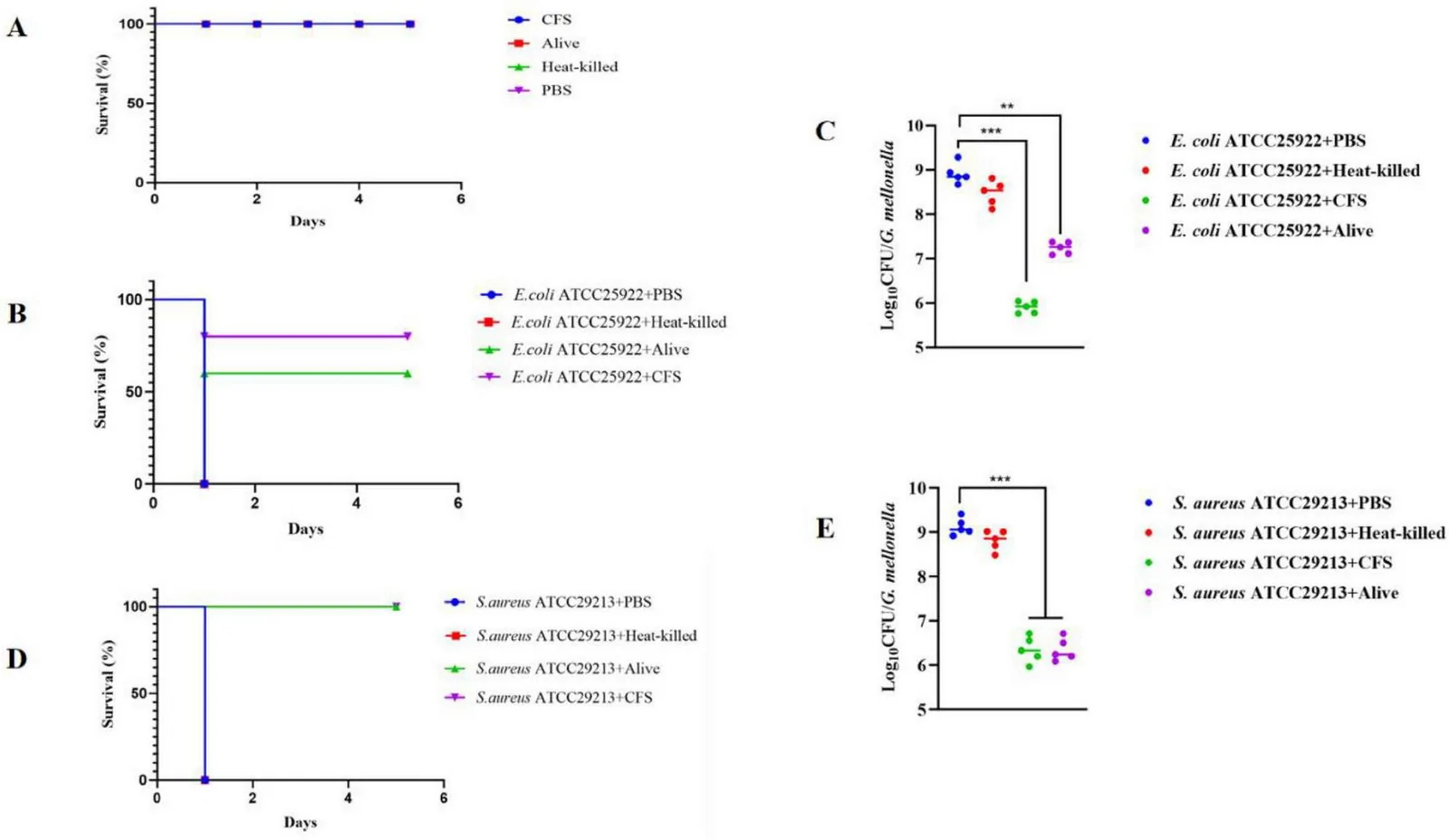
Toxicity and therapeutic effect of *VTC3-*KO *S. cerevisiae* against *G. mellonella*. **(A)** Survival curves after a single injection of live yeast, heat- inactivated yeast, or cell-free supernatant (CFS) (*n* = 5). **(B)** Survival curves of larvae infected with *E. coli* treated with *VTC3-*KO *S. cerevisiae* or CFS. **(C)** *E. coli* loads in larval homogenates on day 5. **(D)** Survival curves of larvae infected with *S. aureus* treated with *VTC3*-KO *S. cerevisiae* or CFS, the CFS-treated group showed 100% survival at all recorded time points, identical to the live-cell group; therefore, the CFS survival curve completely overlapped with the Alive curve. **(E)** *S. aureus* loads in larval homogenates on day 5. Data are shown as mean ± standard deviation (SD). **p < 0.01; ***p < 0.001.

#### 3.8.2 *VTC3*-KO S. cerevisiae protects against *S. typhimurium*-induced enteritis in mice

To evaluate the *in vivo* antibacterial effect of *VTC3*-KO *S. cerevisiae* against *S. typhimurium*, a mouse infection model was established. A 7- day safety test showed that mice treated with *VTC3*-KO *S. cerevisiae* or BY4743 exhibited body weight gain similar to that in the 0.9% saline group, confirming low toxicity (Figure 8A). On day 7, mice were infected with S. typhimurium and monitored for 5 days. The results showed that *VTC3*-KO *S. cerevisiae* treatment significantly alleviated body weight loss caused by infection (Figure 8B) and increased the 10-day survival rate (Figure 8C), demonstrating significant protection.

**FIGURE 8:**
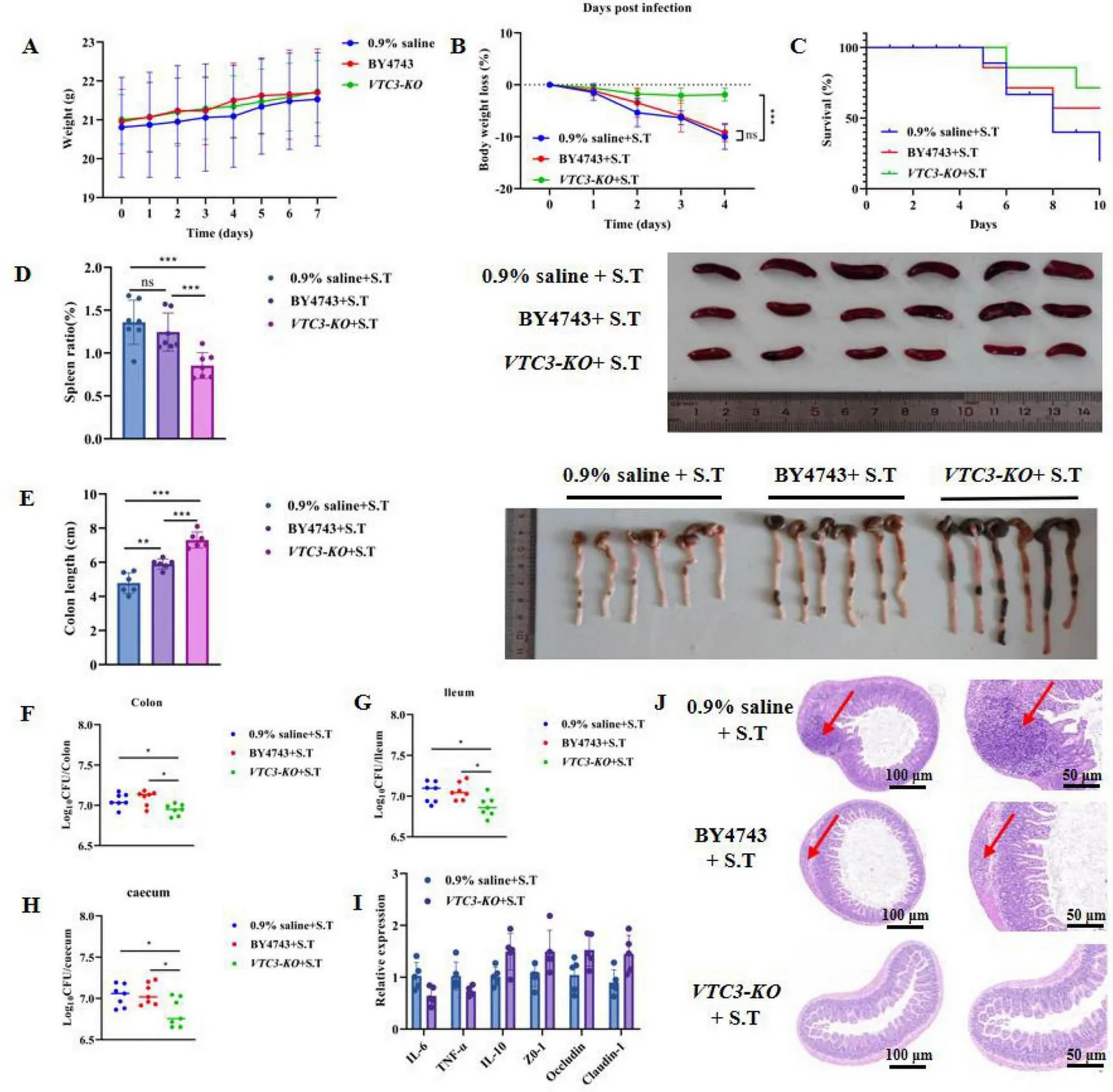
*VTC3-*KO *S. cerevisiae* protects mice against *S. typhimurium* infection. **(A)** Safety test: body weight of three groups over 7 days (*n* = 12). **(B)** *VTC3-*KO *S. cerevisiae* alleviated body weight loss after infection (*n* = 6). **(C)** Survival rate after infection. **(D,E)** *VTC3-*KO *S. cerevisiae* inhibited splenomegaly, colonic shortening, and cecal congestion. **(F–H)** *VTC3-*KO *S. cerevisiae* reduced bacterial loads in the ileum, colon, and cecum. **(I)** Real-time PCR results. **(J)** Hematoxylin and eosin staining; red arrows indicate inflammatory infiltration; scale bars: 100 μm and 50 μm. Data are shown as mean ± standard deviation (SD); *p < 0.05; **p < 0.01; ***p < 0.001; ns, not significant.

On day 5 post- infection, the 0.9% saline and BY4743 groups showed splenic congestion and enlargement, increased spleen index, colonic shortening and congestion, and cecal sac atrophy (Figures 8D,E). In contrast, the *VTC3-*KO *S. cerevisiae* group had nearly normal spleen and colon morphology. Bacterial load results showed that *VTC3-*KO *S. cerevisiae* treatment significantly reduced the load of *S. typhimurium* in the ileum, colon, and cecum (Figures 8F–H).

To further investigate the effect of *VTC3-*KO *S. cerevisiae* on the inflammatory response, the expression levels of key cytokines in mouse ileal tissue were measured. Compared with 0.9% saline, *VTC3-*KO *S. cerevisiae* treatment significantly downregulated levels of the proinflammatory cytokines IL- 6 and TNF- α and upregulated that of the anti- inflammatory cytokine IL- 10 (Figure 8I), shifting the intestinal microenvironment toward an anti- inflammatory state. H&E staining showed that the 0.9% saline and BY4743 groups exhibited obvious inflammatory cell infiltration, epithelial cell shedding, and vascular congestion in the ileum, whereas the *VTC3-*KO *S. cerevisiae* group maintained intact villus structure, no tissue edema, and minimal inflammatory cell infiltration (Figure 8J), indicating effective protection of the intestinal barrier. Moreover, microbial infection disrupts intestinal barrier integrity and increases permeability. We further examined the expression of tight junction proteins. The results showed that *VTC3-*KO *S. cerevisiae* significantly upregulated occludin, claudin-1, and ZO-1 compared with the saline control (Figure 8I). These results suggest that *VTC3-*KO *S. cerevisiae* alleviates *S. typhimurium*-induced intestinal damage by inhibiting inflammation and enhancing the intestinal epithelial barrier.

### 3.9 Metabolomic analysis shows that the metabolites with antibacterial activity in CFS are mainly organic acids

First, unsupervised principal component analysis (PCA) was used to visualize metabolomic differences. The results showed that the CFS of *VTC3-*KO and BY4743 were clearly separated along the PC1/PC2 dimensions (Figure 9A), indicating that the two strains possessed distinctly different metabolic profiles. Supervised partial least squares-discriminant analysis (PLS-DA) further confirmed this intergroup separation trend (Figure 9B) with robust model parameters (R^2^ > 0.99 and Q^2^ > 0.95 after sevenfold cross-validation), verifying the reliable predictive performance of the model. The Human Metabolome Database (HMDB) is currently the largest and most comprehensive species-specific metabolite database.

**FIGURE 9:**
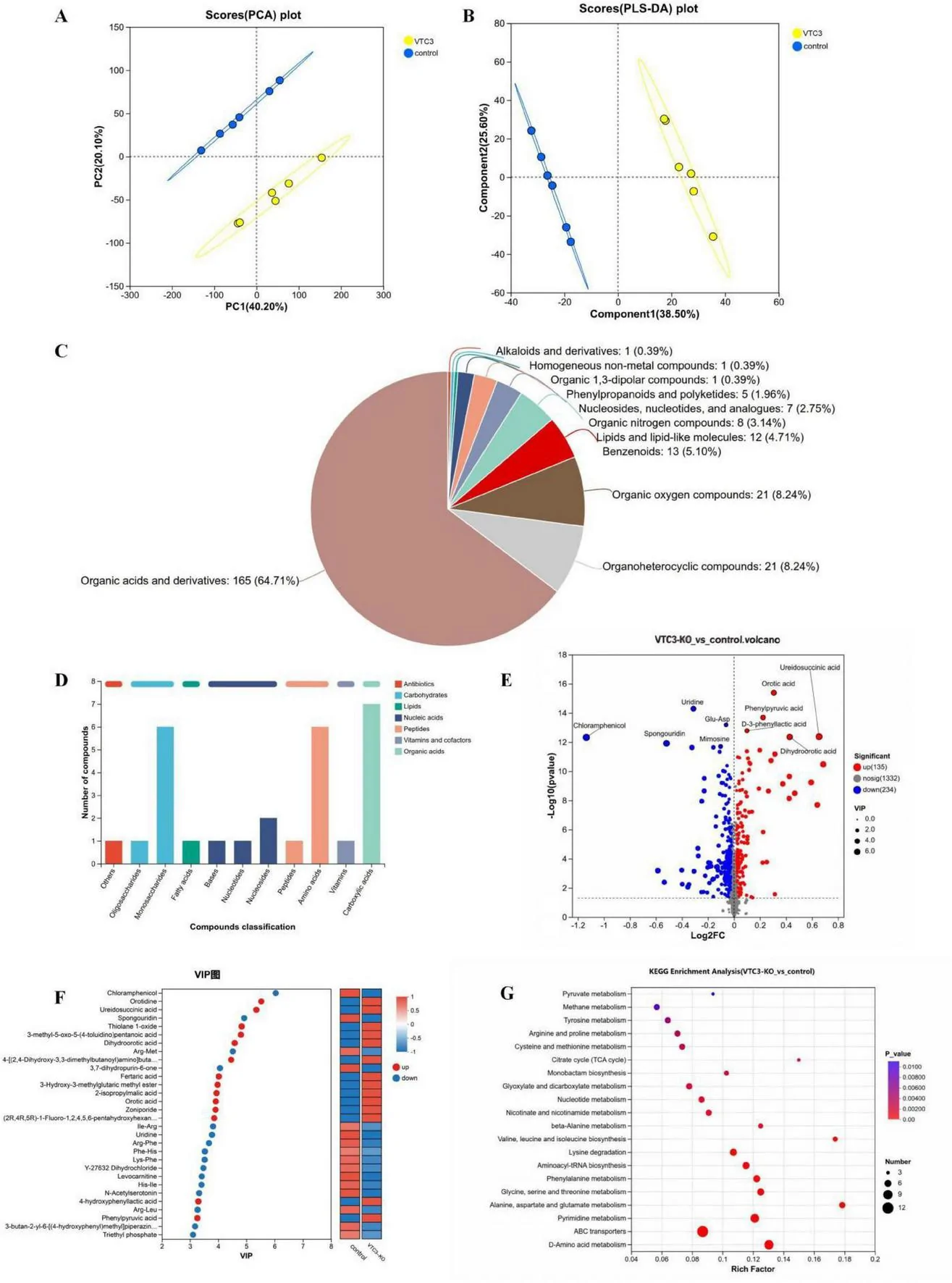
Untargeted metabolomics analysis revealed significant enrichment of organic acids in *VTC3-*KO *S. cerevisiae*. **(A)** Principal component analysis (PCA) and **(B)** Partial least squares-discriminant analysis (PLS-DA) score plots; **(C)** annotation results of the Human Metabolome Database (HMDB); **(D)** KEGG subclass classification; **(E)** volcano plot; **(F)** variable importance in projection plot; **(G)** KEGG enrichment analysis.

Based on the annotation results of the HMDB database, among the 165 significantly differential metabolites, organic acids and their derivatives accounted for 64.71% (Figure 9C). The Kyoto Encyclopedia of Genes and Genomes Compound Database (KEGG) collects small molecules, biopolymers, and other chemical substances related to biological systems. In contrast, according to the classification statistics of KEGG compound subcategories, amino acid/peptide derivatives ranked as the second most abundant metabolite category (Figure 9D). In this study, differential metabolites were strictly screened by combining three criteria: variable importance in the projection value of orthogonal PLS-DA ≥ 1, absolute value of fold change ≥ 1, and *p-*value < 0.05. A total of 369 significantly differentially expressed metabolites were identified from the corresponding volcano plot results (Figure 9E), among which 234 were downregulated and 135 were upregulated. The majority of the 135 upregulated metabolites were organic acids, including orotic acid, dihydroorotic acid, ureidosuccinic acid, phenylpyruvic acid, and D-3-phenyllactic acid. The variable importance in the projection score plot (Figure 9F) also independently verified the above key differential metabolites. Many of these metabolites have been proven to possess antibacterial, antioxidant, or immunomodulatory activities, which explains the molecular mechanism underlying the enhanced antibacterial activity of the CFS of the *VTC3-*KO strain at the chemical level.

In addition, KEGG pathway enrichment analysis (hypergeometric test, *p* < 0.05 after Benjamini–Hochberg correction) revealed significant enrichment of amino acid metabolism, pyrimidine metabolism, aminoacyl-tRNA biosynthesis, nicotinate/nicotinamide metabolism, and ABC transporter pathways (Figure 9G). Among them, the D-amino acid metabolism pathway showed a high enrichment level. The α-keto acid products generated by this pathway can directly enter the tricarboxylic acid cycle and gluconeogenesis pathway, both of which serve as core hubs for the interconversion of upregulated organic acids (citric acid, isocitric acid, α-ketoglutaric acid, succinic acid, fumaric acid, malic acid, and oxaloacetic acid). In conclusion, *VTC3* knockout can reshape the carbon metabolic flux, shifting it toward the organic acid biosynthesis pathway, thereby expanding the reservoir of metabolites with antibacterial activity.

### 3.10 *VTC3*-KO *S. cerevisiae* exerts antibacterial effects by producing active metabolites

To elucidate the antibacterial mechanism involved, stability and activity assays were performed using *E. coli* ATCC25922 and *S. aureus* ATCC29213 under various physicochemical and enzymatic treatments. As shown in Figure 10A, the antibacterial activity of *VTC3-*KO *S. cerevisiae* CFS was highly pH- dependent. Clear inhibition was observed under acidic conditions (pH 2–4); however, this effect was attenuated or lost at neutral to alkaline pH (6–8). Enzyme treatment (Figure 10B) showed that proteases such as pepsin, trypsin, and papain did not significantly alter the antibacterial activity, indicating that the active substance is not proteinaceous. Heat stability experiments (Figure 10C) showed that after treatment at 60°C, 80°C, or even 100°C, the antibacterial activity remained essentially stable, with no significant decline.

**FIGURE 10:**
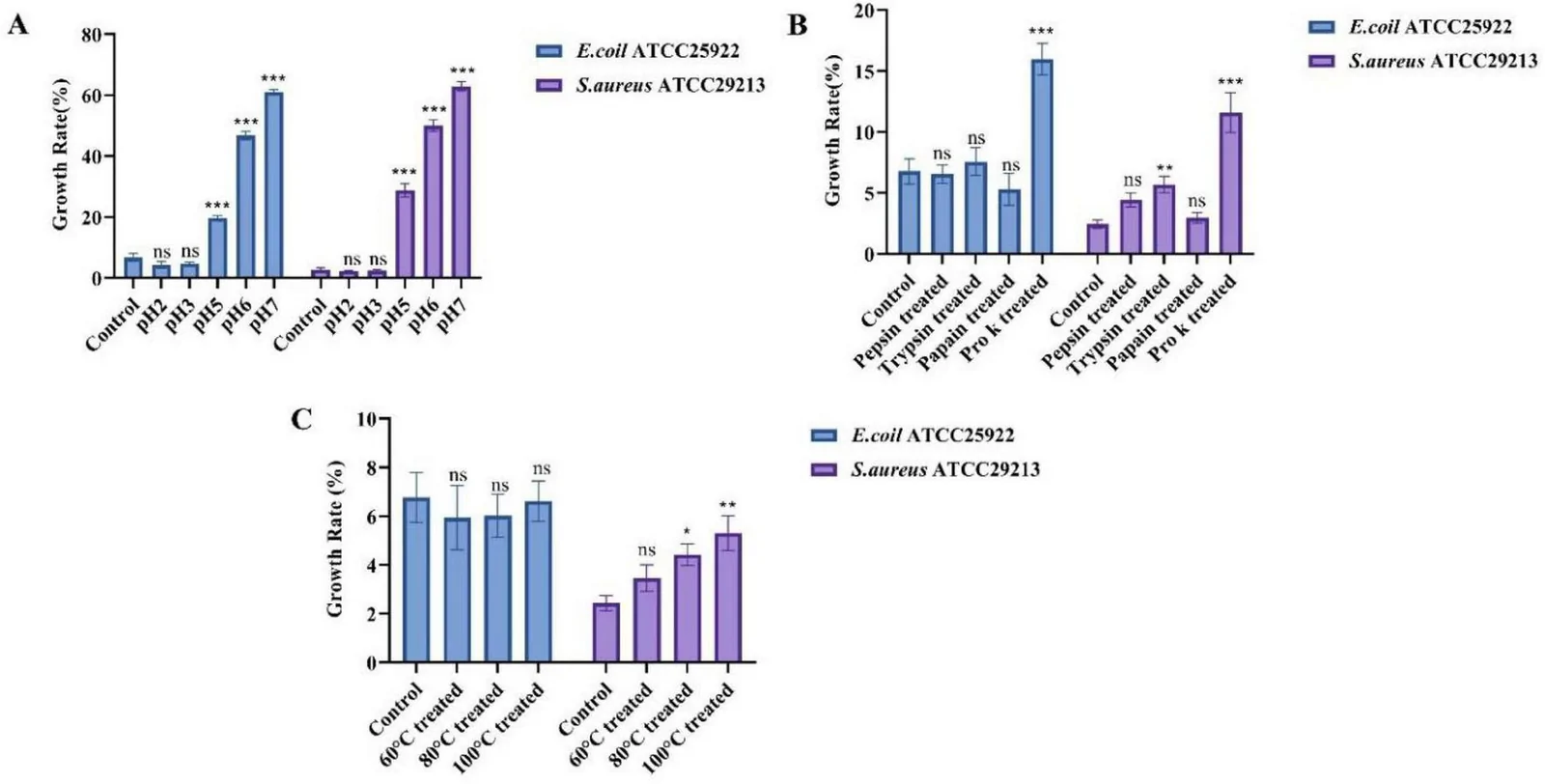
Metabolites produced by *VTC3-*KO *S. cerevisiae*. **(A)** pH neutralization experiment. **(B)** Enzyme treatment experiment. **(C)** Heat-treatment experiment. Data are shown as mean ± standard deviation (SD). *p < 0.05; **p < 0.01; ***p < 0.001; ns, not significant.

## 4 Discussion

Multidrug-resistant bacterial infections have emerged as a major challenge to global public health, with a continued increase in the associated disease burden and healthcare costs (Antimicrobial Resistance Collaborators, 2022). As resistance mechanisms continue to evolve, the clinical efficacy of conventional antibiotics has markedly declined, leading to increasing limitations in standard therapeutic regimens. In particular, current anti-infective strategies frequently fail to achieve satisfactory therapeutic outcomes against pathogens with complex resistance phenotypes and rapidly disseminating resistance genes, (Faridgohar, 2020; Sy et al., 2022; Javan et al., 2025). Clinically, biofilm-mediated persistent infections and sustained intestinal colonization by MDR bacteria constitute core drivers of failed anti-infective therapy and recurrent infections (McArthur, 2019). Although antibiotics eliminate pathogenic bacteria, they simultaneously disrupt the intrinsic intestinal microecological balance, further facilitating the selection and enrichment of drug-resistant strains, thereby creating a vicious cycle of “infection-antibiotic intervention-exacerbated drug resistance.” Thus, it is crucial to develop novel, safe antibacterial agents adaptable to drug-resistant phenotypes to provide sustainable solutions for the prevention and treatment of drug-resistant bacterial infections.

Probiotics, as a class of live microbial preparations, provide a new strategy for the prevention and control of drug-resistant bacterial infections by modulating host immunity, competing for ecological niches, and secreting bioactive metabolites (Kaźmierczak-Siedlecka et al., 2020). Compared with bacterial probiotics, yeasts exhibit higher tolerance to harsh gastrointestinal conditions, characterized by the presence of gastric acid and bile salts (de Melo Pereira et al., 2018). Moreover, they are not inhibited by the vast majority of clinical antibiotics, conferring the potential for combined application with antibiotics (Pais et al., 2020; Ding et al., 2025). Using a high- throughput knockout screening system, we systematically investigated the antibacterial activity 505 of *VTC3*-KO *S. cerevisiae* and examined the underlying mechanisms for the first time.

Growth curve assays confirmed that *VTC3* knockout did not induce growth defects in yeast, and the growth curves of *VTC3*- KO and BY4743 were nearly superimposable. *In vitro* antibacterial assays demonstrated that the CFS of *VTC3*- KO markedly suppressed the growth of MDR pathogens, including *E. coli* mcr- 1, MRSA, and *A. baumannii* 21- 1. Antibacterial spectrum analysis revealed that the residual growth rate of *E. coli* mcr- 1 treated with CFS from *VTC3*- KO was only 21.04% ± 0.39%, whereas that of *A. baumannii* 21- 1 decreased to as low as 1.74% ± 0.03%. Its antibacterial effect was significantly superior to those of the BY4743 and *S. boulardii* control groups. These observations indicate that deletion of the *VTC3* gene confers an enhanced antibacterial phenotype to S. cerevisiae. However, the *VTC3* gene is primarily involved in the synthesis and transport of vacuolar polyphosphate (PolyP). As a gene governing phosphate homeostasis, it does not directly participate in organic acid biosynthesis. This suggests that the phosphate- PolyP signaling network represents a previously underappreciated regulatory node that indirectly remodels global carbon metabolic flux in yeast. This finding provides new perspectives for the metabolic engineering of antibacterial yeasts, distinct from conventional carbon metabolism-related targets.

Bacterial pathogenicity relies on their growth capacity and is closely associated with virulence factors such as biofilm formation and adhesion. Biofilms represent a critical contributor to persistent chronic infections and antibiotic resistance, and biofilm development proceeds through four sequential stages: attachment to biotic or abiotic surfaces, microcolony aggregation, biofilm maturation, and bacterial dispersal (Le et al., 2025; Patra et al., 2025). Probiotics can attenuate the pathogenicity of pathogenic bacteria by intervening at any of these stages (Dsouza et al., 2024). Disrupting biofilm structure and eradicating sessile cells are critical to prevent colonization (Liu X. et al., 2023). The findings of this study demonstrated that the CFS of *VTC3*- KO *S. cerevisiae* inhibited biofilm formation of the tested Gram-positive and Gram-negative bacteria as well as MDR strains in a dose-dependent manner. Upon treatment with 90% (v/v) *VTC3*- KO CFS, the biofilm inhibition rates of *E. coli* ATCC 25922, *E. coli* mcr- 1, *S. aureus* ATCC 29213, MRSA, and *K. pneumoniae* ATCC 700603 reached 86.31%, 85.29%, 83.61%, 87.44%, and 83.05%, respectively. There is a complex interaction between adhesion, hydrophobicity, and EPS production, affecting biofilm formation and environmental adaptation (St John et al., 2020). Furthermore, bacterial surface hydrophobicity and EPS production are positively correlated with adhesion; higher hydrophobicity and EPS lead to higher adhesion (Aboelnaga et al., 2024). The results of this study revealed that treatment with 50% (v/v) *VTC3*- KO CFS reduced the adhesion capacity of *E. coli* by 29.45% and that of *S. aureus* by 37.89%. Their cell- surface hydrophobicity decreased by 13.05% and 16.28%, respectively, whereas EPS production was lowered by 22.61% and 16.57%. Collectively, these data indicate that metabolites from culture supernatant primarily interfere with the early adhesion step of biofilm development. Within the intestinal microenvironment, such activity can attenuate pathogen attachment to the intestinal epithelium and reduce the colonization potential of pathogenic bacteria, thereby preventing infection onset. Nevertheless, in this study, we only investigated the inhibitory effect of CFS on the transition of planktonic bacteria to biofilms; its capacity to eradicate fully mature biofilms remains unevaluated. Mature biofilms possess a dense matrix barrier that impedes the penetration of bioactive metabolites. Further studies should establish *in vitro* models of mature biofilms to achieve a comprehensive evaluation of the antibiofilm efficacy of this yeast strain. Another limitation is that *BY4743* CFS was not included as a parallel control in the biofilm and virulence assays (Figures 3, 4). Consequently, a direct comparison of the strain-specific effects between *BY4743* and *VTC3-KO* CFS in these specific endpoints was not possible. Future studies incorporating parallel *BY4743* CFS controls are warranted to fully dissect the differential antibacterial mechanisms between the parental and knockout strains.

Probiotics must survive transit through the entire gastrointestinal tract to exert their antibacterial effects (Hill et al., 2014). Gastric acid and small-intestinal bile salts constitute two major physiological barriers that probiotics need to overcome before reaching the human intestine. The pH in the human stomach generally ranges from 1.5 to 3.0, and bile salt concentrations in the small intestine vary between 0.03% and 0.3% (Whitehead et al., 2008; Bustos et al., 2018). In this study, *in vitro* simulated gastrointestinal fluid assays demonstrated that *VTC3*-KO *S. cerevisiae* exhibited a survival rate of 82.13% after 1 h incubation in artificial gastric fluid at pH 3.0, and the survival rate remained above 75% at 3 h. Following 4 h incubation in artificial intestinal fluid containing 0.3% bile salts, the strain maintained a survival rate of 65.17%. These findings confirm that *VTC3*-KO *S. cerevisiae* possesses favorable tolerance toward both conditions. Although the strain showed good tolerance to simulated gastric and intestinal fluids, these static assays do not reflect the dynamic process of intestinal colonization. Long-term colonization capacity, spatial distribution along the gastrointestinal tract, and persistence over time were not evaluated. Future studies should include time-course colonization assays *in vivo*, using selective plating or fluorescent labeling, to determine whether the strain can stably colonize the gut and maintain its antibacterial phenotype under continuous flow and competitive microbiota pressure.

Auto- aggregation and coaggregation are key properties for probiotics to survive in the complex intestinal environment and exert antibacterial functions (Morrissey and Patel, 2025). The former enhances community function through improved colonization and synergistic metabolism, whereas the latter directly interferes with pathogen adhesion and proliferation (López-García et al., 2024). Auto-aggregation is an important screening criterion for probiotics, facilitating stable colonization in the gut, whereas enhanced coaggregation enables physical “aggregation-clearance” mechanisms to inhibit pathogen growth, representing an important competitive exclusion mechanism (Sivignon et al., 2015). In the static *in vitro* assays performed in this study, the *VTC3*-KO strain exhibited a 16.48% increase in autoaggregation capacity compared with BY4743. Its coaggregation rates against *E. coli* and *S. aureus* were elevated by 26.31% and 15.4%, respectively. It is speculated that *VTC3* knockout alters the physicochemical components on the yeast cell surface, thereby modifying its aggregation phenotype.

It should be objectively noted that these aggregation experiments were performed under static *in vitro* conditions. The *in vivo* intestinal tract; however, is subject to complex dynamic physiological conditions including peristalsis, digesta flow, and mucus layer shear. Static *in vitro* results cannot fully recapitulate the real intestinal microenvironment. Future studies may employ *in vitro* intestinal microfluidic devices to introduce fluid shear stress and further evaluate the aggregation behavior and intestinal retention capacity of this strain. In summary, the favorable gastrointestinal tolerance, autoaggregation and coaggregation properties enable *VTC3*-KO S*. cerevisiae* to establish a spatial barrier to inhibit adhesion and colonization of pathogenic bacteria while generating an intestinal 588 microenvironment conducive to the function of antibacterial metabolites.

Live yeast–pathogen coculture assays further confirmed that the viable *VTC3*-KO yeast possesses the capacity to directly suppress the proliferation of pathogenic bacteria. After 16 h of coculture, the inhibition rates exerted by *VTC3*- KO against *E. coli* ATCC 25922, *S. aureus* ATCC 29213, and *K. pneumoniae* ATCC 700603 reached 49.21%, 89.81%, and 45.77%, respectively. Comparable inhibitory effects were still maintained at 24 h of incubation, directly demonstrating that the viable yeast can exert antibacterial activity.

Animal models represent an indispensable tool for validation of the preclinical efficacy of probiotics. In the present study, *G. mellonella* larvae were employed for preliminary *in vivo* activity screening. Compared with mammals, this model possesses conserved innate immune pathways and is suitable for rapid *in vivo* evaluation of antibacterial agents (Tsai et al., 2016). The *G. mellonella* model possesses an immune system that is structurally and functionally analogous to mammalian innate immunity (Mendoza Barker et al., 2024). Its cost-effectiveness, simple rearing, conserved innate immune pathways, and viability at 37 °C make it an attractive and flexible platform for studying host responses to a wide range of pathogens. Indeed, this model has been successfully applied to dissect the virulence mechanisms of pathogenic yeasts (e.g., *Candida*, *Cryptococcus*, *Trichosporon*) and filamentous fungi (e.g., *Aspergillus*, *Mucorales*) (Jemel et al., 2020). A *S. typhimurium*-induced mouse enteritis model was further adopted for verification at the mammalian level. *S. typhimurium* is a major cause of foodborne diarrhea worldwide, and studies in infection models can directly guide clinical treatment and vaccine development (Zhang et al., 2020). Results from *G. mellonella* suggested that heat-killed *VTC3-*KO yeast conferred no *in vivo* protective effects. Conversely, viable yeast cells and their CFS improved the survival rate of infected hosts and reduced *in vivo* bacterial loads. These observations indicate that the *in vivo* protective efficacy of this strain relies on the metabolic and secretory activities of live cells, rather than the structural components of yeast cells per se (Sun et al., 2021). In the mouse infection model, preliminary safety experiments confirmed that intragastric administration of *VTC3*- KO *S. cerevisiae* for seven consecutive days did not trigger abnormal body-weight loss in mice. Following *S. typhimurium* challenge, the *VTC3*- KO pretreatment group exhibited significant mitigation of infection- induced body- weight loss and prolonged 10-day survival. The spleen is an important immune organ, and colon length measurement is a key, intuitive pathological indicator in enteritis models (Tsai et al., 2016). At the pathological level, *VTC3*- KO treatment alleviated infection-provoked splenomegaly as well as colonic shortening and hyperemia. Tissue colony counting revealed markedly reduced burdens of *S. typhimurium* in the ileum, colon, and cecum. qPCR and H&E-stained tissue sections demonstrated that treatment with the *VTC3*-KO strain downregulated the transcription of proinflammatory cytokines, including IL6 and TNF- α, while upregulating that of the anti- inflammatory cytokine IL- 10 in ileal tissues. Meanwhile, it significantly increased the expression of tight- junction proteins occludin, claudin1, and ZO1. Consistently, H&E staining showed intact intestinal villus architecture and remarkably diminished inflammatory- cell infiltration in the *VTC3*- KO group. Collectively, these findings suggest that *VTC3*- KO *S. cerevisiae* exerts dual protective effects *in vivo*. It directly inhibits the proliferation and colonization of pathogenic bacteria via secreted bioactive metabolites, thereby attenuating pathogen invasion into intestinal tissues. Further, it modulates host inflammatory responses and preserves the integrity of the intestinal epithelial barrier to reduce bacterial translocation. Notably, in the mouse experiments in this study, we adopted a prophylactic intragastric regimen in which yeast was administered prior to pathogen challenge. This setting better mimics the scenario for preventing intestinal colonization by MDR bacteria. Nevertheless, established infections are encountered more frequently in clinical practice. Further studies are therefore required to include therapeutic groups with post-infection administration in order to examine the efficacy of this strain against established intestinal infections. In addition, the SPF-grade mice used in this experiment possess a relatively simple gut microbiota background that differs from the complex human intestinal microecology. Human microbiota638 associated mouse models could be adopted to more faithfully reflect the functional performance of this strain within complex microbial communities.

Existing studies have shown that *S. cerevisiae* can secrete organic acids, such as lactic acid (Cong et al., 2025), fatty acids (Liu et al., 2019), and polyamines (Bianchi et al., 2019), to inhibit the growth of pathogenic bacteria. Notably, Offei et al. (2019) demonstrated that high acetic acid production underlies the distinct antibiotic potency of *S. boulardii*, a mechanism that parallels our observation with the *VTC3*- KO strain. This suggests that organic acid- mediated antibacterial activity represents a conserved trait among certain yeast strains. Although the CFS components of *S. cerevisiae* are complex and the chemical structures of some metabolites remain unclear, untargeted metabolomics is still an effective research method for analyzing a large number of metabolites in complex systems (Zhou et al., 2020). We performed analyses combining physicochemical treatment assays and untargeted LC- MS/MS-based metabolomics to decipher the material basis underlying the antibacterial activity of *VTC3*- KO *S. cerevisiae*. pH- gradient experiments revealed that the antibacterial activity of CFS exhibited obvious pH- dependence: robust activity was observed under acidic conditions at pH 2–4, whereas the inhibitory effect was markedly attenuated or even abolished when pH increased to the neutral- to- alkaline range of pH 6–8. Treatment with pepsin, trypsin, proteinase K, and papain did not substantially alter the antibacterial activity of the supernatant, demonstrating that the active constituents are not proteins or polypeptides. Antibacterial activity was largely retained after 1 h of thermal treatment at 60 °C, 80 °C, and even 100 °C, indicating that the bioactive metabolites possess thermostability. These physicochemical properties are highly consistent with those of small-molecule organic acids. For metabolomic analysis, the raw dataset was sequentially subjected to filtering, missing-value imputation, normalization, and log-transformation, ultimately yielding 919 and 782 high-quality feature peaks, respectively. Combining univariate *t*-test and multivariate orthogonal PLS-DA (variable importance in projection > 1), a total of 369 differential metabolites were screened from 2955 annotated metabolites, among which 135 were upregulated and 234 were downregulated. Organic acids and their derivatives accounted for as high as 64.71 % of the upregulated metabolites, including orotic acid, dihydroorotic acid, ureidosuccinic acid, phenylpyruvic acid, D-3-phenyllactic acid, and other small molecules previously reported to exert antibacterial and immunomodulatory activities. KEGG pathway enrichment analysis revealed significant enrichment of the D-amino acid metabolic pathway. Studies have confirmed that nanomolar concentrations of D-leucine, D-methionine, D-tyrosine, and D-tryptophan can inhibit the biofilm formation of *Bacillus subtilis* (Kolodkin-Gal et al., 2010) and *S. aureus* (Hochbaum et al., 2011). The key rate-limiting enzyme D-amino acid oxidase (Kobayashi, 2019) can catalyze D-amino acids to produce α-keto acids, and the products directly enter the tricarboxylic acid cycle, linking carbon metabolic flux and the acid production process. Mechanistic studies suggest that the antibacterial activity of *VTC3*-KO *S. cerevisiae* is mediated by the production of heat- stable, non- proteinaceous, pH- dependent metabolites. The pH- dependent characteristics indicate that these metabolites may have enhanced activity or stability under acidic conditions, which is highly consistent with the properties of bioactive compounds such as organic acids, secondary metabolites, and redox- active small molecules.

*VTC3* encodes a component of the vacuolar membrane transport complex involved in polyphosphate (PolyP) synthesis and transport (Liu W. et al., 2023), and PolyP plays a central role in cellular phosphate homeostasis and stress responses (Kulakovskaya et al., 2021; Deitert et al., 2025). We hypothesize that *VTC3* deletion perturbs phosphate metabolism and energy balance, redirecting metabolic flux toward fermentation and increasing the secretion of short- chain organic acids (acetic, succinic, and citric acid) (Kulakovskaya et al., 2023). These organic acids lower the environmental pH. Many bacteria, particularly those that grow under neutral or slightly alkaline conditions (such as *S. aureus* and *P. aeruginosa*), are highly sensitive to low pH. Acidic conditions disrupt membrane potential and inhibit key enzyme activities, thereby effectively suppressing bacterial growth (Christaki et al., 2020). Yeasts generally have greater acid- tolerance than several bacteria, thus possessing a competitive advantage. This finding links the *VTC3*/PolyP pathway to the antibacterial metabolic phenotype of yeast, providing a new target for metabolic engineering to enhance probiotic functions. However, the specific metabolic changes and the mechanism by which the PolyP pathway alterations precisely regulate the synthesis of antibacterial substances require further investigation through metabolomics and gene expression profiling.

This study has several limitations. First, untargeted metabolomics only identified broad categories of organic acids, and individual active molecules were not purified. Consequently, whether the antibacterial effect arises from a single acid or from synergistic interactions remains unclear, and the downstream regulatory network linking *VTC3*/PolyP to organic acid production requires further elucidation. Second, the causal role of specific metabolites was not validated, and long-term resistance development was not assessed; thus, the possibility of resistance cannot be excluded. Third, although the activity is pH-dependent, its stability and efficacy under physiological pH, as well as potential effects on host tissues, were not evaluated. Fourth, *in vitro* assays used single-pathogen cultures and did not include complex microbiota, mucus, or fecal matrices. Fifth, *in vivo*, the prophylactic model, lack of colonization dynamics and persistence data, and unassessed translocation risk limit translation. Sixth, although the same batch of YPD medium was used for both *BY4743* and*VTC3-KO* cultures, a no-culture YPD control incubated for the same duration was not included. Therefore, we could not perform background subtraction to fully exclude the possible presence of natural YPD degradation products. Finally, long-term safety, chronic toxicity, immunogenicity, horizontal gene transfer, genetic stability, and the effective dose window remain to be determined.

Future studies should therefore focus on: (1) targeted isolation and characterization of specific antibacterial metabolites, followed by validation with purified compounds; (2) long-term resistance induction assays under subinhibitory concentrations; (3) pH-controlled *in vivo* studies or protective delivery strategies to maintain organic acid activity at target sites; (4) dynamic colonization and competition assays in complex microbiota models; and (5) chronic safety assessments, including toxicity, immunogenicity, and horizontal gene transfer, to define the therapeutic dose window for clinical translation; (6) Future studies incorporating a no-culture YPD blank control and baseline subtraction are warranted to fully validate the specific metabolic generation profile of the yeast strains.

## 5 Conclusion

In conclusion, in the present study, we demonstrate that *VTC3* gene knockout endows *S. cerevisiae* with excellent anti- pathogenic capacity and probiotic properties, *in vitro* and *in vivo. In vitro* assay findings verified that CFS derived from *VTC3-*KO yeast exerts broad-spectrum inhibitory effects against MDR pathogens, suppresses biofilm formation in a dose-dependent manner, and reduces bacterial adhesion, cell-surface hydrophobicity, and EPS production. The *VTC3-* KO strain exhibits robust tolerance to gastrointestinal stress and significantly enhanced autoaggregation and coaggregation abilities. Furthermore, *G. mellonella* and *S. typhimurium*-induced enteritis mouse models validated its *in vivo* protective functions, including improved host survival, reduced bacterial burdens in intestinal tissues, decreased intestinal inflammatory damage, and preserved intestinal barrier integrity. Despite these findings, several scientific challenges remain to be addressed prior to practical clinical application. Future investigations will focus on the isolation and identification of core active substances, the dissection of the molecular regulatory network underlying *VTC3*-mediated carbon metabolism remodeling, functional validation under complex gut microbiota conditions, and systematic evaluation of the biosafety of the engineered strain.

## Data Availability

The datasets presented in this study can be found in online repositories. The names of the repository/repositories and accession number(s) can be found below: https://ngdc.cncb.ac.cn/bioproject/browse/PRJCA067538 (no.OMIX018074), OMIX 018074.
